# The Diet after Operations on the Stomach and Intestines

**Published:** 1903-06-13

**Authors:** 


### THE DIET AFTER OPERATIONS ON THE STOMACH AND INTESTINES.

IT is needless to insist on the supreme importance of careful dieting after operations on the abdominal viscera; especially when the stomach or bowel have been opened or sewn up, as in gastrotomy for ulcer or malignant disease, gastro-intestinal anastomosis, partial gastrectomies and so forth.

Take for example the case of a perforated gastric or duodenal ulcer, in which an operation has been performed and the ulcer removed or closed. The patient is put back to bed, suffering not only from the shock due to the severe operation but also from some inevitable peritonitis. It is obvious that his life depends on the skill and care with which he is nursed and fed. The surgeon has to face a dilemma: the vitality of the patient is brought very low, and nothing remedies this so quickly and well as judicious feeding by the mouth; but on the other hand the stomach must be kept quiet and free from the strain of digestion. No definite rule can be laid down; some experienced operators begin almost at once to give small quantities of liquid nourishment by the mouth, whereas others wait for five or six days. Probably we shall do best to take a middle course; and the following programme may be carried out in most cases without much fear.

In the first place give no actual nourishment by the mouth for two or three days; sips of hot water may be given from the first, to allay the thirst and dryness of the tongue and mouth, and if there is much collapse a teaspoonful of brandy may be taken also, but it is better not to give the latter if one can help it; and luckily there are other ways of stimulating which are very efficacious, such as injections of strychnine or ether hypodermically, or enemas of brandy and hot saline solution. An ounce of brandy with six ounces of hot water and half a teaspoonful of salt make an excellent stimulating enema, which may be repeated every three or four hours if necessary. For actual *food* the patient must rely on rectal feeding. This may support life for a period of weeks or even months, but it cannot do so satisfactorily. Physiology teaches us that no peptonisation worth considering takes place in the large intestine; but this is not necessary, for many forms of ordinary proteid are capable of being absorbed, even in the rectum, without digestion occurring at all. In fact, peptones, especially pancreatised milk, are sometimes irritating to the lower bowel, and are not retained so well as undigested food. Raw beef juice and eggs are absorbed very well, especially if a little salt is added; solutions of carbodydrates (starch, etc., as in arrowroot, sago-water, etc.), and especially dilute solutions of grape-sugar or dextrine are also absorbed without difficulty. It may be noted that solution of grape-sugar is absorbed most rapidly at the strength of 0·5 per cent. It is stated that a little red wine, especially claret, is useful in rectal feeding, as it aids the retention of the nutrient enema.

It is best to vary the food, sometimes giving a couple of eggs beaten up in 250 grms. of milk with a grm. of salt and a wineglassful of claret, or half an ounce of beef-juice or bovinine, the yolk of an egg, half an ounce of brandy, and six or seven ounces of 10 per cent. solution of grape sugar; or the formula of Leube (mentioned in Van Valzah's and Nisbet's "Diseases of the Stomach") of 150 grms. of beef-pulp, 50 grms. of the pulp of fresh pancreas of the cow, with 100 grms. of tepid water. Washing out the rectum with warm water is very important, and this should be done twice a day, before the nutrient enema. The food should be slowly injected by long tube and funnel, not by syringe.

As to frequency, every six hours is probably often enough. Dr. Bevan, of Chicago (*Journal of American Medical Association*, January 24th, 1903), recommends 12 ozs. of normal saline solution to be used alternately with the "nutrients."

By these means it will be possible to keep the patient fairly well nourished for two or three days; and then, if all is going well, very carefully arranged mouth feeding may be begun.

Here again we are helped by physiological experiments, especially those on man himself. During digestion in the stomach acid gastric juice is secreted; and this may be just enough to digest the food, or there may be great excess. There is also movement of the stomach, a "churning" movement and an occasional peristaltic wave. We ought to give food which will not excite much secretion in the organ or cause much movement. Stimulating substances, such as condiments (salt, pepper, etc.) and especially the *extractives* of meat cause a rapid secretion of gastric juice and, apparently, stimulate the walls of the stomach to contract. Therefore these should be strictly forbidden; and all kinds of beef teas, especially the concentrated essences which contain so large a percentage of extractives should be absolutely avoided for the first ten days or fortnight. It has been found by giving "test" meals to healthy men, and obtaining the contents of the stomach by passing a tube and "washing out" at intervals afterwards, that raw eggs pass into the duodenum very quickly, in fact are probably digested in the intestines. They are consequently excellent for our purpose. But it is best to begin with "albumen water" made by beating up the "whites" of two eggs with half a pint of water. This may be given in table-spoonful doses every hour, the dose being gradually increased, and rectal feeding being, of course, also continued. Then, say on the fourth or fifth day, peptonised milk or gruel may be given, also in small doses. The milk should be diluted with water or barley-water and a dessert-spoonful of brandy may be mixed with it now and then. The amount of stimulant given must depend on the patient's condition. After this ordinary milk (diluted) with sometimes a little lime water, and eggs beaten up (yolks and whites) with milk and water, may be taken. During these critical days the patient, more especially if a woman, will be constantly demanding "a cup of tea." On the sixth or seventh day this may generally be given, but it should be weak, freshly made, and *without* much milk. It is a mistake to suppose that tea is rendered more digestible by adding a quantity of milk; the tannin and essential oils hinder the digestion of the milk, the milk does not do away with the action of these substances.

On the eighth or ninth day some "pounded" fowl may be given. This is made by pounding the white meat, carefully separated from fat, gristle, etc., in a mortar and adding milk to make it into a "mash." On the ninth or tenth day some soft milk pudding (sago, or semolina, etc.) may be given, and a few days after, minced chicken, mild unseasoned soups and sweetbread may be taken, and lightly boiled eggs, with thin slices of bread and butter—the bread not to be new.

Luckily, very little decomposition goes on naturally in the stomach, but it is so important to avoid this that farinaceous foods, such as milk puddings, rice, sago, etc., should be given very sparingly for the first two or three weeks.

Fish should not be given before the third week, and the first (because the most easily digested) should be whiting. Beef teas should, for reasons given before, be avoided until the end of the second or third week.

To briefly summarise:—Rectal feeding for first few days: Nutrient enemas of eggs, milk, barley-water, solutions of grape sugar, raw beef juice, bovinine, salt, claret, brandy, etc., the food being varied from time to time; frequency, about every four to six hours.

Mouth feeding: Albumen water at first, followed by peptonised gruel, peptonised milk diluted, eggs (raw), solution of grape sugar, ordinary milk, pounded fowl, sago or semolina pudding, etc., minced chicken, lightly-boiled eggs, toast or bread and butter, tender minced mutton, etc., somewhat in the above order.

